# Bias in measurement of autism symptoms by spoken language level and non-verbal mental age in minimally verbal children with neurodevelopmental disorders

**DOI:** 10.3389/fpsyg.2022.927847

**Published:** 2022-07-29

**Authors:** Shuting Zheng, Aaron Kaat, Cristan Farmer, Audrey Thurm, Catherine A. Burrows, Stephen Kanne, Stelios Georgiades, Amy Esler, Catherine Lord, Nicole Takahashi, Kerri P. Nowell, Elizabeth Will, Jane Roberts, Somer L. Bishop

**Affiliations:** ^1^Department of Psychiatry and Behavioral Sciences, Weill Institute for Neurosciences, University of California, San Francisco, San Francisco, CA, United States; ^2^Feinberg School of Medicine, Northwestern University, Chicago, IL, United States; ^3^Neurodevelopmental and Behavioral Phenotyping Service, National Institute of Mental Health, Bethesda, MD, United States; ^4^Department of Pediatrics, University of Minnesota, Minneapolis, MN, United States; ^5^Masonic Institute for the Developing Brain, University of Minnesota, Minneapolis, MN, United States; ^6^Center for Autism and the Developing Brain, Weill Cornell Medical College, White Plains, NY, United States; ^7^Offord Centre for Child Studies, McMaster University, Hamilton, ON, Canada; ^8^UCLA Semel Institute for Neuroscience & Human Behavior, Center for Autism Research and Treatment, David Geffen School of Medicine, University of California, Los Angeles, Los Angeles, CA, United States; ^9^Thompson Center for Autism and Neurodevelopmental Disorders, University of Missouri, Columbia, MO, United States; ^10^Department of Health Psychology, University of Missouri, Columbia, MO, United States; ^11^Department of Psychology, University of South Carolina, Columbia, SC, United States

**Keywords:** autism symptoms, measurement invariance, language level, non-verbal mental age, ADOS

## Abstract

Increasing numbers of children with known genetic conditions and/or intellectual disability are referred for evaluation of autism spectrum disorder (ASD), highlighting the need to refine autism symptom measures to facilitate differential diagnoses in children with cognitive and language impairments. Previous studies have reported decreased specificity of ASD screening and diagnostic measures in children with intellectual disability. However, little is known about how cognitive and language abilities impact the measurement of specific ASD symptoms in this group. We aggregated a large sample of young children (N = 1196; aged 31–119 months) to examine measurement invariance of ASD symptoms among minimally verbal children within the context of the Autism Diagnostic Observation Schedule (ADOS) Module 1. Using confirmatory factor analysis (CFA) and moderated non-linear factor analysis (MNLFA), we examined how discrete behaviors were differentially associated with the latent symptom domains of social communication impairments (SCI) and restricted and repetitive behaviors (RRB) across spoken language levels and non-verbal mental age groupings. While the two-factor structure of SCI and RRB held consistently across language and cognitive levels, only partial invariance was observed for both ASD symptom domains of SCI and RRB. Specifically, four out of the 15 SCI items and one out of the three RRB items examined showed differential item functioning between children with “Few to No Words” and those with “Some Words”; and one SCI item and one RRB item showed differential item functioning across non-verbal mental age groups. Moreover, even after adjusting for the differential item functioning to reduce measurement bias across groups, there were still differences in ASD symptom domain scores across spoken language levels. These findings further underscore the influence of spoken language level on measurement of ASD symptoms and the importance of measuring ASD symptoms within refined spoken language levels, even among those with minimal verbal abilities.

## Introduction

Evidence of social communication impairments (SCI) and restricted and repetitive behaviors (RRB) is required for a diagnosis of autism spectrum disorder (ASD) (American Psychiatric Association., 2013). However, symptoms in these two domains occur commonly in children with a range of other neurodevelopmental disorders (NDDs), greatly complicating differential diagnosis (Grzadzinski et al., 2011; Hepburn and Moody, 2011; Bishop et al., 2019; Lord and Bishop, 2021). Differential diagnosis of ASD is especially challenging in the context of intellectual disability (ID) (Moss and Howlin, 2009; Thurm et al., 2019). By definition, children with ID exhibit delays in social communication relative to same-aged peers (American Psychiatric Association., 2013), and they often present with RRBs (Evans and Gray, 2000; Moss et al., 2009; Burbidge et al., 2010; Wolff et al., 2012; Hoch et al., 2016). Not surprisingly, therefore, children with lower IQ/mental age often receive elevated scores on ASD symptom measures, regardless of whether they ultimately receive a clinical diagnosis of ASD (Havdahl et al., 2016).

Decreased specificity (i.e., higher false positive rate) of commonly used diagnostic instruments such as the Autism Diagnostic Interview-Revised (ADI-R) (Lord et al., 1994; Rutter et al., 2005) and Autism Diagnostic Observation Schedule (ADOS)/ADOS-2 (Lord et al., 2000, 2012) is particularly pronounced among children with very low mental ages and/or non-verbal IQ below 50 (Risi et al., 2006). Thus, the authors have cautioned against interpreting scores in children with non-verbal mental ages below 15–18 months for the ADOS and below 24 months for the ADI-R (Lord et al., 1994, 2012). Nevertheless, these measures are still widely applied in clinical and research samples of children with very low levels of language and cognitive abilities. Especially as DSM-5 now explicitly allows for the diagnosis of ASD with a range of other conditions, a growing number of children with known genetic diagnoses, many of whom have severe to profound intellectual disability (ID), are being referred for assessment of ASD (Hepburn and Moody, 2011; King et al., 2014; Richards et al., 2015; Abbeduto et al., 2019).

Understanding how cognitive and/or language ability affects the measurement of ASD symptoms has implications for clinical practice and research involving children with ASD, other NDDs, and/or genetic conditions (Thurm et al., 2019). Inaccurate diagnosis may lead to delayed or inappropriate clinical services, and in the research context, presents a serious threat to the validity of ASD case vs. control studies. Further, if measures systematically provide higher or lower symptom scores for individuals with certain characteristics (regardless of ASD status), the score differences will fail to represent true differences in abilities/impairments across groups. Numerous studies have established that both language and cognitive ability influence the manifestation of ASD-related symptoms, which in turn may affect accuracy of classifications yielded by ASD symptom measures in certain groups (Risi et al., 2006; Corsello et al., 2007; Gotham et al., 2007; Kim and Lord, 2012; Hus et al., 2013; Havdahl et al., 2016). However, there is much less work on how specific aspects of ASD symptom measurement are affected by developmental and/or language level. This information is needed to increase precision of measurement of ASD symptoms in the context of extreme developmental variability that characterizes NDD clinical and research populations.

Examining measurement invariance (MI)/differential item functioning (DIF) across groups defined based on certain characteristics is one way to advance ASD measurement in this area. MI refers to “the situation in which scales provide the same results across different samples or populations” (Zedeck, 2014, p. 211), which is a critical property of measures that allows factor scores to be compared meaningfully across groups. MI is often tested in a stepwise fashion with increasingly strict standards for equivalence. Specifically, MI is commonly tested across three levels of equivalence: (1) configural invariance of the number of factors and loading pattern, (2) metric invariance of the factor loadings, which reflect the strength of the associations between the items and the factors (i.e., latent constructs), and (3) scalar invariance of the intercepts, which indicates the means of item scores across groups were reflective of means of the latent construct. Adequate MI is established by demonstrating that constraints on each of the parameters described above do not significantly worsen model fit. For more information on MI and differential item functioning, please see Widaman and Reise (1997); Teresi and Fleishman (2007), and Bauer et al. (2020).

In recent years, multiple studies on MI/DIF have been carried out with different ASD symptom measures, including the Childhood Autism Rating Scale (CARS) (Schopler et al., 1988, 2010), ADOS (Lord et al., 2000, 2012), Social Responsiveness Scale, and ADI-R (Constantino, 2005; Constantino and Gruber, 2012). These studies primarily focused on the effects of race/ethnicity, sex/gender and chronological age on scores (ADOS: Harrison et al., 2017; Ronkin et al., 2021; Burrows et al., 2022; Kalb et al., 2022; CARS: Stevanovic et al., 2021; SRS and ADI-R: Frazier and Hardan, 2017), with a few studies also investigating MI across groups with or without ID (Sturm et al., 2017; Dovgan et al., 2019). While these studies provided preliminary evidence that ASD symptom measures should take the impact of cognitive abilities into account, understanding of how cognitive or language abilities influence the measurement of specific ASD symptoms is still limited. Thus, the current study chose to focus on children with developmental delays to clarify the impact of finer divisions of cognitive and language abilities on the measurement of ASD symptom domains within this population. This information is necessary to improve the measurement of ASD symptoms within this special group, wherein differential diagnosis of ASD is especially challenging.

The ADOS is one of the most commonly used measures in the diagnostic assessment of ASD. Module 1 is designed for individuals with chronological age over 31 months who are not yet using flexible phrase speech; thus, children receiving Module 1 present with clinically significant delays in language and/or overall development. However, even among this group, there is substantial variability in age and non-verbal cognitive ability, as well as in expressive language ability (i.e., from no word approximations or words to beginning use of multiple word combinations). Therefore, examining MI of the latent constructs of ASD symptom domains in the context of the ADOS Module 1 provides a unique opportunity to elucidate the impact of mental age and spoken language level on the measurement of ASD symptoms in children with developmental delays.

## Materials and methods

### Participants

Data for the current analyses were aggregated from multiple sites to obtain a large sample of children who received ADOS Module 1 as part of a comprehensive diagnostic evaluation. Participants were included in the current analysis if they: (1) were between 31 and119 months at the time of ADOS administration; (2) had undergone a comprehensive diagnostic evaluation to determine a best-estimate diagnosis of ASD or another non-ASD NDD; (3) had complete data on the selected items from ADOS Module 1; (4) received a developmental/cognitive assessment at the time of the ADOS Module 1 administration; and (5) had cognitive assessment information available for the calculation of non-verbal age equivalents. This resulted in 1043 children with ASD and 153 without ASD from seven sites (see Supplementary material for details about data sources and sample aggregation). Table 1 shows the demographic characteristics of the study sample.

**TABLE 1:** Sample characteristics.

		Non-ASD (*n* = 153)	ASD (*n* = 1043)
ADOS Module 1 language levels	Few to No word	49(32%)	474 (45.5%)
	Some words	104 (68%)	569 (54.5%)
Non-verbal mental age*^a^*	Below 24 months	62 (40.5%)	244 (23.4%)
	24 months and above	91 (59.5%)	799 (76.6%)
Sex	Male	104 (68.0%)	847 (81.2%)
	Female	49 (32.0%)	196 (18.8%)
Race	White	109 (71.2%)	688 (66.0%)
	Black	15 (9.8%)	116 (11.1%)
	AAPI	4 (2.6%)	71 (6.8%)
	AIAN	1 (0.7%)	5 (0.5%)
	Other	18 (11.8%)	116 (11.1%)
	Missing	6 (3.9%)	47 (4.5%)
Ethnicity	Hispanic	19 (12.4%)	117 (11.2%)
	Non-Hispanic	117 (76.5%)	868 (83.2%)
	Missing	17 (11.1%)	58 (5.6%)
Primary diagnosis*^b^*	Down Syndrome	37 (24.2%)	
	Language Disorders	21 (13.7%)	
	ID unknown etiology	15 (9.8%)	
	Fragile X Syndrome	11 (7.2%)	
	Williams Syndrome	7 (4.6%)	
	Global Developmental Delay	5 (3.3%)	
	Others	3 (2.0%)	
	Not Specified	54 (35.3%)	
		**N, Mean (SD), Range**	**N, Mean (SD), Range**
	Age	153, 46.15 (13.94), 31-115	1043, 62.11 (22.32), 31-119
	Non-verbal mental age	153, 25.89 (8.01), 6-58	1043, 32.61 (14.29), 2-104
	Non-verbal IQ	133, 59.85 (21.44), 13-133	1037, 55.66 (20.52), 2-144
	Verbal IQ	132, 52.70 (20.63), 11.83-110	1025, 38.26 (20.26), 3-103

^*a*^NVMA < 15months: N_(*non–ASD*)_ = 8 (5.2%), N_(*ASD*)_ = 26 (2.5%); 15 months ≤ NVMA < 18 months: N_(*non–ASD*)_ = 12 (7.8%), N_(*ASD*)_ = 38 (3.6%).

^*b*^Other primary diagnoses for the non-ASD group include one Cerebral Palsy, one Behavioral Disorder, and one genetic syndrome. Cases from all data sources have clinical best-estimate diagnoses of ASD and non-ASD, but some did not have primary diagnosis information available.

### Measures

The Autism Diagnostic Observation Schedule (Lord et al., 2000, 2012) is a standardized, semi-structured observational assessment designed to elicit social communication and restricted and repetitive behaviors associated with a diagnosis of ASD. It was designed to accommodate the assessment of ASD symptoms across language levels, with developmentally appropriate activities and codes organized into Modules (Lord et al., 2000, 2012). In the current analysis, we only included participants who were administered Module 1, designed for individuals who do not yet use flexible phrase speech. Consistent with scoring conventions, item scores of 0,1, and 2 were included in the analysis as they were, scores of 3 were converted to 2s for analysis, and scores of 8 (“Not applicable”) and 9 (“Unknown”) were converted to 0s.

As reflected in DSM-5 diagnostic criteria for ASD (American Psychiatric Association., 2013), previous factor analyses of the ADOS have consistently identified two core symptom domains of SCI and RRB (Gotham et al., 2007, 2008; Huerta et al., 2012; Harrison et al., 2017). Therefore, the current analyses focused on a subset of items mapping onto the two latent constructs of interest, SCI and RRB. Items on play (Section C) and other abnormal behaviors (Section E) were excluded. We also excluded the following items, as they were later added in the ADOS-2 and therefore missing for older cases who received ADOS-G: B13a *Amount of Social Overtures/Maintenance to Attention: Examiner*; B13b *Amount of Social Overtures/Maintenance to Attention: Parent/Caregiver*; B14 *Quality of Social Response*; B15 *Level of Engagement*; *B16 Overall Quality of Rapport*. Item A6 *Use of Another’s Body* was excluded as, unlike the other SCI items, it reflects the presence of abnormal behavior rather than the absence of developmentally expected behavior. For RRB, we excluded items that were dependent on sufficient spoken language to exhibit the abnormality (A3 *Intonation of Vocalizations*, A4 *Immediate Echolalia*, A5 *Stereotyped/Idiosyncratic Use of Words or Phrases*). We also excluded Item D3 *Self-Injurious Behavior* due to an extremely low rate of endorsement (<9% endorsing 1s or 2s). In total, 15 items assessing SCI and three items assessing RRB were included in the analyses (see Table 2).

**TABLE 2:** ADOS Module 1 Items included in the analyses.

	Item level	Item description
Social communication impairments	A2	*Frequency of Spontaneous Vocalization Directed to Others*
	A7	*Pointing*
	A8	*Gestures*
	B1	*Unusual Eye Contact*
	B2	*Responsive Social Smile*
	B3	*Facial Expressions Directed to Others*
	B4	*Integration of Gaze and Other Behaviors During Social Overtures*
	B5	*Shared Enjoyment in Interaction*
	B6	*Response to Name*
	B7	*Requesting*
	B8	*Giving*
	B9	*Showing*
	B10	*Spontaneous Initiation of Joint Attention*
	B11	*Response to Joint Attention*
	B12	*Quality of Social Overtures*
Repetitive behaviors and restricted interests	D1	*Unusual Sensory Interests in Play Material/Person*
	D2	*Hand and Finger and Other Complex Mannerisms*
	D4	*Unusually Repetitive Interests or Stereotyped Behaviors*

For detailed item description and scoring instruction of each item, please refer to ADOS Module 1 scoring protocol (Lord et al., 2012).

*Spoken Language Level*. We derived the language level classification based on Item A1 “*Overall Level of Non-Echoed Spoken Language*” from the ADOS Module 1. Consistent with instructions for use of the revised algorithms (Gotham et al., 2007), participants who received scores of 3 or 4 were assigned to “Few to No words” and participants who received scores of 0, 1, or 2 were assigned to “Some words” group. The validity of these spoken language groups is further supported by previous studies showing differences between “Few to No Words” and “Some Words” on other measures of expressive language and cognitive ability (Bal et al., 2016; Mazurek et al., 2019).

*Non-verbal mental age.* Participants included in the aggregated dataset were administered at least one measure of cognitive ability based on site-specific protocols and/or clinician judgment about the developmentally appropriate test: the Mullen Scales of Early Learning (MSEL; (Mullen, 1995), the Differential Ability Scales (DAS) (Elliott, 2007), and/or the Merrill-Palmer Scales of Development (Roid and Sampers, 2004). The MSEL was used for 89% of the non-ASD sample and 75% of the ASD sample. For each participant, a non-verbal mental age was derived based on averaging available age equivalents from the non-verbal subtests. For those who received the MSEL, the age equivalents from the Fine Motor and Visual Reception subscales were averaged to represent NVMA (see Bishop et al., 2011; Farmer et al., 2016).

We dichotomized the NVMA variable to NVMA under 24 months vs. NVMA of 24 months and above for both practical and theoretical reasons: (1) given different tests were administered across sites, the binary categories will achieve more reliable grouping by avoiding the point estimates of the NVMA; (2) the cut point at 24 months allows sufficient sample sizes in both groups; (3) moreover, 24 months is an age at which children would be expected to use phrase speech in typical development (Sheldrick et al., 2019); thus, children with a non-verbal mental age above 24 months who receive Module 1 (rather than Module 2 or 3) show evidence of a discrepancy between their non-verbal mental age and their spoken language level. Therefore, we might expect that items developed for children with a very low spoken language level (i.e., language abilities characteristic of children under 24 months) might function differently in those with higher NVMA.

*Best Estimate Diagnosis of ASD.* All participants underwent multi-disciplinary evaluations by experienced clinicians and/or researchers who had established and maintained research reliability on the ADOS/ADOS-2. Best-estimate clinical diagnoses of ASD or the absence of ASD (i.e., Non-ASD) were determined based on all available information, including parent interviews of developmental history and direct observation of ASD symptoms (including the ADOS), as well as tests of cognitive and adaptive functioning.

### Statistical analyses

#### Confirmatory factor analyses

Separate CFA with two factors (SCI and RRB; see Table 2 for ADOS Module 1 item mapping onto the two factors) were conducted across two spoken language level groups and two NVMA groups, respectively, to examine configural invariance (i.e., the number of factors and loading pattern) (Widaman and Reise, 1997). Factor analyses were conducted in *Mplus* with WLSMV estimator for ordered categorical variables. The chi-square statistics, comparative fit index (CFI), the root mean square error of approximation (RMSEA) and its 90% confidence interval (CI), and the standardized root mean square residual (SRMR) were examined for CFA model fit, with CFI larger than 0.95, RMSEA and SRMR smaller than 0.08 indicating a good fit (Hu and Bentler, 1999).

### Moderated non-linear factor analysis

Once configural invariance was established through the CFA across NVMA and spoken language level groups, we proceeded to examine higher levels of structural validity testing of the two latent constructs across covariate groupings of interests (both of which were analyzed using effects coding): spoken language level (i.e., −1 = Few to No words vs. 1 = Some words) and developmental level (i.e., NVMA: −1 = under 24 months vs. 1 = 24 months and above). Moderated Non-linear Factor Analysis (MNLFA) is similar to both the multiple-group CFA and the multiple-indicator multiple-cause (MIMIC) methods for evaluating measurement invariance, but it extends both to multiple groups, categorical or count data, and the inclusion of multiple grouping variables at the same time. In the MNLFA model, MI/DIF is viewed as a form of parameter moderation; and thus, tested in the model for statistical significance as moderators of factor and item parameters. That is, moderation of the intercepts would indicate uniform DIF, whereas moderation of the factor loadings would indicate non-uniform DIF. We recommend that interested readers refer to Bauer (2017) for more details. Since MNLFA only accommodates unidimensional factor structure, separate analyses were conducted for SCI and RRB. The MNLFA method allows testing of the impact of spoken language levels and NVMA groups at the same time on the mean and variances of latent constructs, as well as their impacts on the intercept and loading of each item on the latent constructs. MNLFA involves an iterative process where each item is tested independently, then the significant (*p* < 0.05) effects are retained and tested simultaneously in one model. Lastly, a final model was estimated using the statistically significant parameters after the Benjamini-Hochberg false discovery rate correction to adjust for multiple comparisons. Moderated item effects were examined and reported to understand the impact of NVMA and spoken language level. The resulting model was then used to estimate the factor scores of the two latent constructs of SCI and RRB.

We employed an updated version of the R package *aMNLFA* Version 1.1.2 (Gottfredson et al., 2019)^1^ to streamline the generation of the *MPlus* codes and automate the process of integrating all effects into one model. We carefully reviewed and modified the automated *MPlus* codes to fit our dataset and research questions.

While there are multiple measures of impact of DIF on the overall measurement of the latent constructs (Meade, 2010), no recommended metric is available for the assessment of overall differential test functioning (DTF) in the context of MNLFA with simultaneous testing of multiple grouping variables. Therefore, to evaluate the differences between DIF-adjusted latent construct scores based on the group-specific information and the factor scores of latent constructs assuming full measurement invariance, we chose to adapt the Root Expected Mean Square Difference (REMSD) which was developed to index subpopulation invariance of linking and equating relationships (Dorans and Holland, 2000). Although MNLFA and equating analyses are distinct, the contrast between group-specific (i.e., with DIF) and overall (i.e., invariant) item parameters in the MNLFA context is comparable to the group-specific and overall equating relationship from which the REMSD statistic was originally derived. The adapted REMSD metric was calculated as the square root of the expected value of squared differences between the DIF-adjusted latent construct scores (FS_*mnlfa*_) and the factor scores assuming full measurement invariance of the item parameters (FS_*FI*_), divided by the standard deviation of the latent factor score (fixed to 1):


$$\text{REMSD}=\sqrt{E\,\left({\left({\mathrm{FS}}_{\mathrm{mnlfa}}-{\mathrm{FS}}_{\mathrm{FI}}\right)}^{2}\right)}/\sigma_{\mathrm{FS}}$$

Further, effect sizes (Cohen’s *d*) were calculated for group comparisons of the latent construct factor scores.

## Results

The majority of the aggregated sample was diagnosed with ASD, which is expected given the data were mostly drawn from autism specialty clinics or research projects focused on ASD (see Table 1). The descriptive statistics showed that, compared to those without ASD, children in the ASD group were more likely to be male (χ^2^ = 13.05, *p* = 0.003), to have “Few to No words” (χ^2^ = 10.02, *p* = 0.002), and to have an NVMA of 24 months and over (χ^2^ = 18.92, *p* < 0.001).

The two-factor structure with SCI and RRB showed a good fit, supporting configural invariance of the ADOS across the two spoken language levels and the two NVMA groups (see Table 3). Table 4 shows item factor loadings onto the two factors of SCI and RRB, respectively, across the two spoken language levels and two NVMA groups.

**TABLE 3:** Fit statistics of two-factor CFA models.

		χ^2^ (df = 134)	CFI	SRMR	RMSEA
Non-verbal mental age	Below 24 months	260.02, *p* < 0.001	0.978	0.057	0.055 [0.045, 0.065]
	24 months and above	428.11, *p* < 0.001	0.975	0.048	0.050 [0.044, 0.055]
Language level	Few to No words	349.48, *p* < 0.001	0.956	0.063	0.055 [0.048, 0.063]
	Some words	345.50, *p* < 0.001	0.978	0.047	0.048 [0.042, 0.055]

**TABLE 4:** Item factor loadings on the two factors from CFA across the groups.

		Non-verbal mental age		Language level	
Factor names		Below 24 months	24 months and above	Few to No words	Some words
Social communication impairment scores	A2	0.91	0.79	0.81	0.79
	A7	0.77	0.70	0.65	0.67
	A8	0.68	0.61	0.60	0.64
	B1	0.91	0.94	0.85	0.99
	B2	0.56	0.55	0.48	0.55
	B3	0.79	0.76	0.73	0.77
	B4	0.88	0.77	0.82	0.78
	B5	0.75	0.65	0.63	0.69
	B6	0.51	0.45	0.41	0.49
	B7	0.73	0.70	0.69	0.67
	B8	0.59	0.55	0.54	0.55
	B9	0.87	0.78	0.85	0.74
	B10	0.78	0.76	0.70	0.75
	B11	0.58	0.54	0.48	0.48
	B12	0.89	0.87	0.87	0.85
Repetitive behaviors and restricted interests scores	D1	0.61	0.62	0.56	0.59
	D2	0.55	0.34	0.56	0.25
	D4	0.57	0.54	0.48	0.62

**FIGURE 1:**
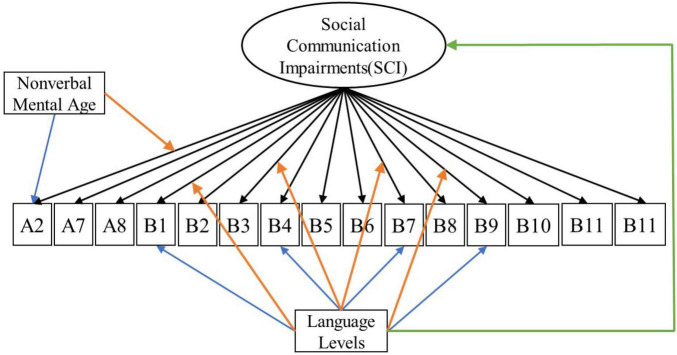
Measurement model for social communication impairments (SCI). Black arrows indicate factor loadings of each item examined on the SCI latent construct. Colored Arrows in the figure showing significant impact of the covariate on the factor and item parameters: (1) Green arrow represents the impact of language level on the mean of the latent construct; (2) Orange arrows represent the impact of covariates (NVMA and language level groups) on the relationships between the item and the latent construct (non-uniform DIF); (3) Blue arrows represent the impact of covariates on the levels of items when the overall level of the latent construct is similar across groups (uniform DIF). For specific item names, please refer to Table 2.

**FIGURE 2:**
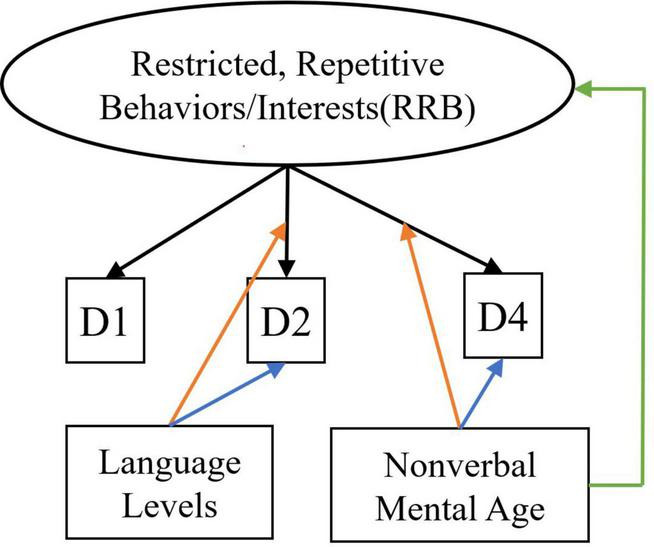
Measurement model for restricted, repetitive behaviors/interests. Black arrows indicate factor loadings of each item examined on the RRB latent construct. Colored Arrows in the figure showing significant impact of the covariate on the factor and item parameters: (1) Green arrow represents the impact of language level on the mean of the latent construct; (2) Orange arrows represent the impact of covariates (NVMA and language level groups) on the relationships between the item and the latent construct (non-uniform DIF); (3) Blue arrows represent the impact of covariates on the levels of items when the overall level of the latent construct is similar across groups (uniform DIF). For specific item names, please refer to Table 2.

For each latent construct, ensuing MNLFAs were conducted separately. For the latent construct of SCI, we observed a significant effect of spoken language level on the measured SCI scores (Estimate = −0.45, SE = 0.034, *p* < 0.001), with individuals with Few to No words showing higher levels of SCI symptoms. Multiple items showed loading and intercept DIF across language levels on the latent construct of SCI, including Unusual Eye Contact, Integration of Gaze and Other Behaviors during Social Overtures, Requesting, and Showing. Only one item, Frequency of Vocalization, showed significant loading and intercept DIF across the NVMA groups on the SCI (see Table 5 upper panel for parameter estimates and Figure 1 for the final SCI measurement model). For the latent construct of RRB, the mean level of measured RRB differed across language levels (Estimate = −0.249, SE = 0.046, *p* < 0.001). There were also loading DIFs of Item “Hand/finger and Other Complex Mannerisms” across spoken language levels and “Unusually Repetitive Interests or Stereotyped Behaviors” across NVMA groups (see Table 5 bottom panel for parameter estimates and Figure 2 for the final RRB measurement models of the two latent constructs). That is, these items show different levels of associations with the latent constructs of SCI and RRB, as well as varying item difficulties. In sum, metric invariance did not hold for several items on both SCI and RRB latent constructs, with subsets of items functioning differently across groups.

**TABLE 5:** Parameter estimates of the resulting MNLFA model.

Parameter type	Variables	Estimate	SE	*p*-value
**Social communication impairments**				
Intercept	ETA	0		
Loading	*Frequency of Vocalization *^a^**	2.794	0.180	<0.001
	*Pointing*	1.624	0.100	<0.001
	*Gestures*	1.229	0.081	<0.001
	*Unusual Eye Contact *^a^**	2.890	0.281	<0.001
	*Responsive Social Smile*	1.006	0.074	<0.001
	*Facial Expressions Directed to Others*	1.835	0.115	<0.001
	*Integration of Gaze and Other Behaviors During Social Overtures *^a^**	2.517	0.163	<0.001
	*Shared Enjoyment in Interaction*	1.463	0.093	<0.001
	*Response to Name*	0.845	0.069	<0.001
	*Requesting *^a^**	1.869	0.127	<0.001
	*Giving*	1.063	0.077	<0.001
	*Showing *^a^**	2.075	0.144	<0.001
	*Spontaneous Initiation of Joint Attention*	1.744	0.110	<0.001
	*Response to Joint Attention*	1.128	0.083	<0.001
	*Quality of Social Overtures*	2.621	0.165	<0.001
Mean Factor	ETA on Language Levels	-0.450	0.034	<0.001
Intercept DIF	*Frequency of Vocalization* on NVMA	−0.369	0.102	<0.001
	*Unusual Eye Contact* on Language Levels	1.595	0.367	<0.001
	*Integration of Gaze and Other Behaviors During Social Overtures* on Language Levels	0.242	0.087	0.005
	*Requesting* on Language Levels	0.067	0.081	0.409
	*Showing* on Language Levels	−0.064	0.106	0.547
Loading DIF	*Frequency of Vocalization* on NVMA	−0.347	0.127	0.006
	*Unusual Eye Contact* on Language Levels	0.780	0.274	0.004
	*Integration of Gaze and Other Behaviors During Social Overtures* on Language Levels	−0.498	0.122	<0.001
	*Requesting* on Language Levels	−0.228	0.102	0.025
	*Showing* on Language Levels	−0.302	0.123	0.014
**Repetitive behaviors and restricted interest**				
Intercept	ETA	0		
Loading	*Unusual Sensory Interests in Play Material/Person*	1.697	0.302	<0.001
	*Hand and Finger and Other Complex Mannerisms *^a^**	0.664	0.114	<0.001
	*Unusually Repetitive Interests or Stereotyped Behaviors *^a^**	1.139	0.178	<0.001
Mean Factor	ETA on Language Levels	−0.258	0.047	<0.001
Intercept DIF	*Hand and Finger and Other Complex Mannerisms* on Language Levels	−0.219	0.069	0.001
	*Unusually Repetitive Interests or Stereotyped Behaviors* on NVMA	0.060	0.080	0.458
Loading DIF	*Hand and Finger and Other Complex Mannerisms* on Language Levels	−0.221	0.106	0.037
	*Unusually Repetitive Interests or Stereotyped Behaviors* on NVMA	−0.413	0.142	0.004

^*a*^Unweighted grand mean of loading across groups. NVMA groups: −1 = under 24 months, 1 = 24 months and above; Language levels: −1 = Few to No Words; 1 = Some Words.

Item-level DIF has moderate to large impact on the score of the two latent constructs: REMSD_*SCI*_ = 0.66 and REMSD_*RRB*_ = 0.74, indicating the need to consider measurement bias in interpreting the measured scores of the two latent constructs. Effect sizes of the DIF-adjusted SCI factor scores indicated that children with “Few to No Words” scored about 1 standard deviation (SD) higher than those with “Some Words” in SCI severity factor scores (Cohen’s *d* = 1.01); similarly, for the RRB factor scores, children with “Few to No Words” scored 0.75 SD higher. On the other hand, small ES were observed for the group comparisons across NVMA of both latent constructs (SCI: ES = 0.34, RRB: ES = 0.27).

## Discussion

The current study was conducted to provide more explicit guidance about how the measurement of ASD symptoms (as indexed by selected items from Module 1 of the ADOS) might be affected by language and developmental level. While decades of research indicate that both language and cognitive ability influence the manifestation and measurement of ASD-related symptoms, there is much less work about specific aspects of ASD symptom measurement that may be problematic when comparing children developmental and spoken language levels. Greater understanding of this issue is important given the extreme developmental heterogeneity that characterizes ASD and NDD clinical and research populations.

Consistent with previous studies of ASD symptom structure, which ultimately informed DSM-5 diagnostic criteria (Huerta et al., 2012; Frazier et al., 2014), findings from the current CFA of the ADOS Module 1indicate two core symptom domains (i.e., SCI and RRB). This structure held across spoken language levels and NVMA groupings, supporting configural invariance of the measure. However, when examining the mean levels of latent constructs for both SCI and RRB, children with “Few to No Words” scored systematically higher (i.e., more impairments) than those with “Some Words”.

When looking at where the ASD symptom measurements showed biases, stricter levels of measurement invariance did not hold at the item level for some items in the MNLFA models for either SCI or RRB latent construct. For the measurement of SCI, loading and intercept DIF was observed for four items across spoken language levels [*Unusual Eye Contact* (B1), *Integration of Gaze and Other Behaviors during Social Overtures* (B4)*, Requesting* (B7), and *Showing* (B9)], and one item [*Frequency of Vocalization*(A2)] across NVMA groups. All four SCI items that showed DIF across spoken language levels involved the use of eye contact with the examiner, highlighting the potential role of spoken language level even when measuring basic non-verbal social communication skills such as eye contact. Even though only a small subset of items (*n* = 5) showed any measurement bias on the latent construct SCI, the DIFs showed impact on the overall latent construct scores, underscoring the need to carefully consider the impact of spoken language levels when making score comparisons between individuals. On the other hand, two out of three RRB items (i.e., *Hand and Finger and Other Complex Mannerisms* and *Unusually Repetitive Interests or Stereotyped Behaviors*) included in the analyses showed bias across either spoken language or NVMA, indicating that the measurement of RRBs with only the three selected items is likely problematic. This is consistent with previous item response theory analyses done with ADOS Modules 3 and 4 (Kuhfeld and Sturm, 2018).

To further understand different levels of autism symptoms across spoken language levels, we compared SCI and RRB factor scores after adjusting for measurement biases identified at the item level, and found that they still differed significantly across spoken language levels, with higher severity scores seen in children with “Few to No Words”. These findings suggest that there are likely true differences in the levels of SCI and RRB symptom severity, as measured using Module 1 of the ADOS, between children with “Few to No Words” vs. “Some Words”. This provides further evidence for the decision to create separate algorithms based on finer language-level divisions within Module 1 (Gotham et al., 2007, 2008). Given that some items on the ADOS Module 1 function differently for children of different spoken language levels, even among those with minimal verbal abilities, clinicians and researchers should follow the algorithm guidelines to derive scores for the two spoken language levels separately and only interpret scores at the domain and scale levels, but not at the item level.

To our knowledge, this study is the first to examine MI of ASD symptoms within children with developmental delays across cognitive and spoken language levels. A deeper understanding of how ASD symptom measurement is affected by developmental level is critical, particularly given increased interests in behavioral phenotyping of rare genetic conditions, many of which are associated with severe to profound ID (Arvio and Sillanpää, 2003; Richards et al., 2015; Abbeduto et al., 2019; Burdeus-Olavarrieta et al., 2021). We focused on ADOS Module 1 to specifically home in on the effects of mental age and expressive language in children with lower cognitive and language abilities. However, this sample does not represent the full range of minimally verbal individuals who have even more severe delays. Importantly, valid administration of the ADOS requires that a child be able to walk, see, and hear at the time of assessment, meaning that it is not even valid for a significant proportion of children with severe to profound ID. Moreover, given the reduced specificity of the measure, the test developers advised against using the ADOS in children with NVMA below 15 months, resulting in very few such cases available for the current analyses: Non-ASD = 8 (5.2%), ASD = 26 (2.5%). Therefore, the present findings have limited applicability to individuals with severe to profound ID and/or sensory and motor impairments, and do not change the recommendation that ADOS scores may not be valid in this group. Yet, the fact remains that clinicians and researchers are increasingly faced with the challenges of assessing ASD symptoms in individuals for whom current measures were not validated, highlighting the need for empirical evidence to measure ASD symptoms validly and reliably in this population. Further, children develop over time and some gain cognitive and language skills as they grow and receive intervention. Thus, future longitudinal studies should examine intra-individual changes as children shift from “Few to No Words” to “Some Words” and/or from lower NVMA group to higher NVMA levels.

The current study represents a first step in understanding ASD symptom measurement for those who are minimally verbal. Even within Module 1, which is already only applicable to children within a relatively narrow developmental range, our findings highlight the need for finer divisions based on spoken language level (e.g., “Few to No Words” and “Some Words”) and/or mental age to optimize measurement of ASD symptoms. Thus, to advance measurement of SCI and RRB in the extremely heterogeneous population of children with neurodevelopmental disorders, the field must work to enhance developmentally appropriate measurement strategies (Bishop et al., 2019). Moreover, it is imperative that clinicians and researchers implement best-practice methods for carefully considering developmental profiles, including cognitive and spoken language levels, in their assessment of ASD-related symptoms and behaviors.

## Data availability statement

Publicly available datasets were analyzed in this study. Data from the Simons Simplex Collections is available to qualified researchers. Approved researchers can obtain the SSC population dataset described in this study by applying at https://base.sfari.org. Data from other sources can be requested by contacting the principal investigators and directors at each site.

## Ethics statement

Ethical review and approval was not required for the study on human participants in accordance with the local legislation and institutional requirements. Written informed consent to participate in this study was provided by the participants or their legal guardian/next of kin.

## Author contributions

SZ and SB conceptualized and designed the study with consultation from AK, CF, and AT. SZ aggregated datasets, conducted statistical analyses, interpreted the results, and drafted the manuscript. AK and CB provided consultation on data analysis and result interpretation. SB together with AK, CF, and AT provided feedback on previous versions of the manuscript. AT, CB, SK, CL, AE, NT, KN, EW, JR, and SB contributed to data collection. SB secured funding for the current study. All authors provided feedback on and approved the final version of the manuscript.

## Footnotes

1
https://github.com/cran/aMNLFA
