# Corrigendum: Bias in measurement of autism symptoms by spoken language level and non-verbal mental age in minimally verbal children with neurodevelopmental disorders

**DOI:** 10.3389/fpsyg.2022.1051464

**Published:** 2022-10-17

**Authors:** Shuting Zheng, Aaron Kaat, Cristan Farmer, Audrey Thurm, Catherine A. Burrows, Stephen Kanne, Stelios Georgiades, Amy Esler, Catherine Lord, Nicole Takahashi, Kerri P. Nowell, Elizabeth Will, Jane Roberts, Somer L. Bishop

**Affiliations:** ^1^Department of Psychiatry and Behavioral Sciences, Weill Institute for Neurosciences, University of California, San Francisco, San Francisco, CA, United States; ^2^Feinberg School of Medicine, Northwestern University, Chicago, IL, United States; ^3^Neurodevelopmental and Behavioral Phenotyping Service, National Institute of Mental Health, Bethesda, MD, United States; ^4^Department of Pediatrics, University of Minnesota, Minneapolis, MN, United States; ^5^Masonic Institute for the Developing Brain, University of Minnesota, Minneapolis, MN, United States; ^6^Center for Autism and the Developing Brain, Weill Cornell Medical College, White Plains, NY, United States; ^7^Offord Centre for Child Studies, McMaster University, Hamilton, ON, Canada; ^8^UCLA Semel Institute for Neuroscience & Human Behavior, Center for Autism Research and Treatment, David Geffen School of Medicine, University of California, Los Angeles, Los Angeles, CA, United States; ^9^Thompson Center for Autism and Neurodevelopmental Disorders, University of Missouri, Columbia, MO, United States; ^10^Department of Health Psychology, University of Missouri, Columbia, MO, United States; ^11^Department of Psychology, University of South Carolina, Columbia, SC, United States

**Keywords:** autism symptoms, measurement invariance, language level, non-verbal mental age, ADOS

In the published article, there was an error in Figure 2 as published. The box connected to D2 incorrectly stated “Nonverbal Mental Age” and the box connected to D4 incorrectly stated “Language Levels.” The corrected Figure 2 and its caption “Measurement Model for Restricted, Repetitive Behaviors/Interests” appear below.

**Figure 2:**
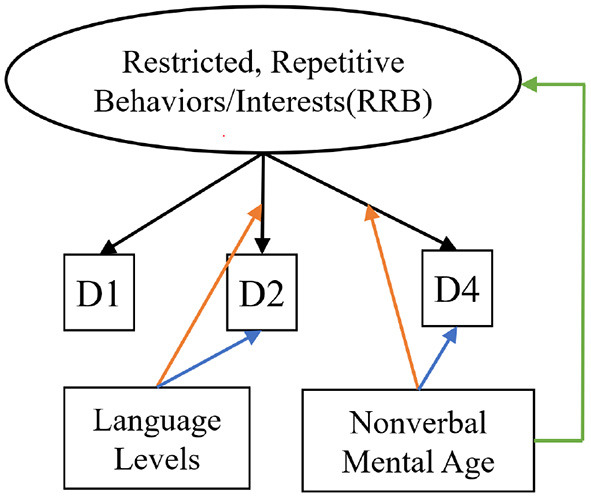
Measurement model for restricted, repetitive behaviors/interests. Black arrows indicate factor loadings of each item examined on the RRB latent construct. Colored Arrows in the figure showing significant impact of the covariate on the factor and item parameters: (1) Green arrow represents the impact of language level on the mean of the latent construct; (2) Orange arrows represent the impact of covariates (NVMA and language level groups) on the relationships between the item and the latent construct (non-uniform DIF); (3) Blue arrows represent the impact of covariates on the levels of items when the overall level of the latent construct is similar across groups (uniform DIF). For specific item names, please refer to Table 2.

In the published article, there was an error in the **Funding** statement. Funding from the Intramural Research Program of the NIMH was erroneously omitted. The correct **Funding** statement appears below.

In the published article there was also an error in Results, Paragraph 3. The item “Unusual Sensory Interests” should be “Unusually Repetitive Interests or Stereotyped Behaviors”. The corrected paragraph is below.

“For each latent construct, ensuing MNLFAs were conducted separately. For the latent construct of SCI, we observed a significant effect of spoken language level on the measured SCI scores (Estimate = −0.45, SE = 0.034, *p* < 0.001), with individuals with Few to No words showing higher levels of SCI symptoms. Multiple items showed loading and intercept DIF across language levels on the latent construct of SCI, including Unusual Eye Contact, Integration of Gaze and Other Behaviors during Social Overtures, Requesting, and Showing. Only one item, Frequency of Vocalization, showed significant loading and intercept DIF across the NVMA groups on the SCI (see Table 5 upper panel for parameter estimates and Figure 1 for the final SCI measurement model). For the latent construct of RRB, the mean level of measured RRB differed across language levels (Estimate = −0.249, SE = 0.046, *p* < 0.001). There were also loading DIFs of Item “Hand/finger and Other Complex Mannerisms” across spoken language levels and “Unusually Repetitive Interests or Stereotyped Behaviors” across NVMA groups (see Table 5 bottom panel for parameter estimates and Figure 2 for the final RRB measurement models of the two latent constructs). That is, these items show different levels of associations with the latent constructs of SCI and RRB, as well as varying item difficulties. In sum, metric invariance did not hold for several items on both SCI and RRB latent constructs, with subsets of items functioning differently across groups.”

The authors apologize for this error and state that this does not change the scientific conclusions of the article in any way. The original article has been updated.

## Funding

This work was supported by grants from the Eunice Kennedy Shriver National Institute of Child Health and Human Development (NICHD; R01HD093012 to SB), and in part by the Intramural Research Program of the NIMH (1ZICMH002961 to AT).

## Publisher's note

All claims expressed in this article are solely those of the authors and do not necessarily represent those of their affiliated organizations, or those of the publisher, the editors and the reviewers. Any product that may be evaluated in this article, or claim that may be made by its manufacturer, is not guaranteed or endorsed by the publisher.

