# Supplementary material for: Bias in measurement of autism symptoms by spoken language level and non-verbal mental age in minimally verbal children with neurodevelopmental disorders
Source: Front Psychol. 2022 Jul 29;13:927847. doi: 10.3389/fpsyg.2022.927847 (PMC9372407; doi:10.3389/fpsyg.2022.927847)
Supplement: Supplementary file 1 [file Table_1.DOCX]

**Supplementary Materials**

# 1. Data Sources

Data were requested and aggregated across seven clinical and research data sources. Inclusion criteria for the current study were sent to the PI/data manager of the databases to request ADOS item-level and best estimate diagnosis data: 1) be aged 31 to 119 months at the time of ADOS administration; (2) have a clinical best-estimate diagnosis of autism spectrum disorder (ASD) or non-ASD; (3) have complete data on the selected ADOS Module 1 items; (4) received developmental/cognitive assessment at the time of the ADOS Module 1 administration; and (5) have cognitive assessment information available for the calculation of nonverbal age equivalents. Specifically, both ASD and non-ASD cases were requested from the data sources of the Center for Autism and Developing Brain (CADB), the National Institute of Mental Health Neurodevelopmental and Behavioral Phenotyping Service (NMIH), and the University of Minnesota (UMN); only ASD cases were available in the Simons Simplex Collection (SSC); and to increase the sample sizes for Non-ASD cases, only non-ASD cases were requested from University of South Carolina (USC), Thompson Center at the University of Missouri, University of California, San Francisco (UCSF). Detailed descriptions of each data source are provided below. The sample sizes across data sources were detailed in Table S1.

**Center for Autism and Developing Brain (CADB)** database at Weill Cornell Medical College is a multi-study clinical research registry of children and adults with or without neurodevelopmental disorders (NDDs). Individuals included in the database were seen as part of regular clinic evaluations or recruited for specific research projects. Diagnoses were made by the clinician's best estimate based on all instrument scores administered as part of the evaluation, but diagnosis of ASD or non-ASD was not contingent on meeting cut-offs on any measure or combination of measures.

**National Institute of Mental Health Neurodevelopmental and Behavioral Phenotyping Service (NMIH)** database is a clinical research database of children and adults with or without NDDs. Diagnoses were made by the clinician's best estimate. Clinicians had access to all instrument scores in determining their diagnoses, but diagnosis of ASD or non-ASD was not contingent upon meeting cut-offs on any measure or combination of measures.

**The University of Minnesota (UMN)** database is a clinical research registry of children and adults with or without neurodevelopmental disorders. Clinicians who had access to all instrument scores made their clinical best estimate diagnoses. However, diagnosis of ASD or non-ASD was not contingent upon meeting cut-offs on any measure or combination of measures.

**Simons Simplex Collection (SSC)** is an open dataset operated by SFARI in collaboration with 12 university-affiliated research clinics, enrolling participants with ASD. The clinics identified and assessed potential SSC participants. Inclusion criteria for the SSC required that probands received a best-estimate clinical diagnosis of ASD and met ASD or autism cut-offs on the ADI-R and ADOS (Fischbach & Lord, 2010; see https://www.sfari.org/resource/simons-simplex-collection/).

**University of South Carolina (USC)** database is a research dataset aggregated across multiple studies on neurodevelopmental disorders, focusing on Fragile X Syndrome and Down Syndrome. The clinical best estimate of the absence of ASD is determined based on comprehensive evaluation data. Only non-ASD cases were requested from the USC.

**Thompson Center at the University of Missouri** database is a clinical research registry of individuals referred for comprehensive evaluation given developmental concerns or individuals who participated in research projects focused on neurodevelopmental disorders. Best estimate diagnoses of ASD and Non-ASD were given by experienced clinicians.

**University of California, San Francisco (UCSF)** Center for ASD and NDD database is a clinical research registry of children and adults with or without neurodevelopmental disorders. Individuals included in the database were seen as part of regular clinic evaluations or recruited for specific research projects. Diagnoses were made by the clinician's best estimate but were not contingent upon meeting cut-offs on any measure or combination of measures.

**Table S1 Sample sizes across data sources by diagnostic groups of ASD and Non-ASD**

| Data Sources | Non-ASD | ASD | Total |
| --- | --- | --- | --- |
| CADB | 48 (31.4%) | 468 (44.9%) | 516 |
| NIMH | 52 (34.0%) | 162 (15.5%) | 214 |
| UMN | 3 (2.0%) | 31 (3.0%) | 34 |
| UCSF | 3 (2.0%) | -- | 3 |
| SSC | -- | 382 (36.6%) | 382 |
| USC | 29 (19.0%) | -- | 29 |
| Thompson Center | 18 (11.8%) | -- | 18 |

**2. Item Endorsement across Groups**

**Table S2 item endorsement rates across developmental groupings**

|  |  | **Nonverbal Mental Age Groups** | | | | **Language Levels** | | | |
| --- | --- | --- | --- | --- | --- | --- | --- | --- | --- |
|  |  | **Below 24 months** | | **24 months and above** | | **Few to No Words** | | **Some Words** | |
|  | Score | % | N | % | N | % | N | % | N |
| A2 | 0 | 14.1% | 43 | 14.4% | 128 | 5.5% | 29 | 21.1% | 142 |
|  | 1 | 26.5% | 81 | 50.0% | 445 | 23.1% | 121 | 60.2% | 405 |
|  | 2 | 59.5% | 182 | 35.6% | 317 | 71.3% | 373 | 18.7% | 126 |
| A7 | 0 | 10.5% | 32 | 13.9% | 124 | 5.9% | 31 | 18.6% | 125 |
|  | 1 | 16.7% | 51 | 38.8% | 345 | 20.7% | 108 | 42.8% | 288 |
|  | 2 | 72.9% | 223 | 47.3% | 421 | 73.4% | 384 | 38.6% | 260 |
| A8 | 0 | 18.6% | 57 | 24.9% | 222 | 16.4% | 86 | 28.7% | 193 |
|  | 1 | 36.9% | 113 | 39.3% | 350 | 39.8% | 208 | 37.9% | 255 |
|  | 2 | 44.4% | 136 | 35.7% | 318 | 43.8% | 229 | 33.4% | 225 |
| B1 | 0 | 14.4% | 44 | 10.3% | 92 | 8.0% | 42 | 14.0% | 94 |
|  | 2 | 85.6% | 262 | 89.7% | 798 | 92.0% | 481 | 86.0% | 579 |
| B2 | 0 | 15.0% | 46 | 22.6% | 201 | 14.5% | 76 | 25.4% | 171 |
|  | 1 | 24.5% | 75 | 28.1% | 250 | 22.9% | 120 | 30.5% | 205 |
|  | 2 | 60.5% | 185 | 49.3% | 439 | 62.5% | 327 | 44.1% | 297 |
| B3 | 0 | 11.4% | 35 | 9.3% | 83 | 5.2% | 27 | 13.5% | 91 |
|  | 1 | 50.3% | 154 | 59.1% | 526 | 52.2% | 273 | 60.5% | 407 |
|  | 2 | 38.2% | 117 | 31.6% | 281 | 42.6% | 223 | 26.0% | 175 |
| B4 | 0 | 17.0% | 52 | 15.3% | 136 | 12.8% | 67 | 18.0% | 121 |
|  | 1 | 30.4% | 93 | 45.8% | 408 | 28.5% | 149 | 52.3% | 352 |
|  | 2 | 52.6% | 161 | 38.9% | 346 | 58.7% | 307 | 29.7% | 200 |
| B5 | 0 | 27.8% | 85 | 32.2% | 287 | 22.2% | 116 | 38.0% | 256 |
|  | 1 | 45.8% | 140 | 46.3% | 412 | 47.4% | 248 | 45.2% | 304 |
|  | 2 | 26.5% | 81 | 21.5% | 191 | 30.4% | 159 | 16.8% | 113 |
| B6 | 0 | 30.4% | 93 | 39.2% | 349 | 31.9% | 167 | 40.9% | 275 |
|  | 1 | 24.8% | 76 | 26.9% | 239 | 23.5% | 123 | 28.5% | 192 |
|  | 2 | 44.8% | 137 | 33.9% | 302 | 44.6% | 233 | 30.6% | 206 |
| B7 | 0 | 36.6% | 112 | 43.8% | 390 | 28.9% | 151 | 52.2% | 351 |
|  | 1 | 43.8% | 134 | 46.5% | 414 | 50.1% | 262 | 42.5% | 286 |
|  | 2 | 19.6% | 60 | 9.7% | 86 | 21.0% | 110 | 5.3% | 36 |
| B8 | 0 | 11.1% | 34 | 10.9% | 97 | 5.2% | 27 | 15.5% | 104 |
|  | 1 | 51.6% | 158 | 56.0% | 498 | 56.4% | 295 | 53.6% | 361 |
|  | 2 | 37.3% | 114 | 33.1% | 295 | 38.4% | 201 | 30.9% | 208 |
| B9 | 0 | 10.5% | 32 | 8.8% | 78 | 5.4% | 28 | 12.2% | 82 |
|  | 1 | 16.0% | 49 | 22.7% | 202 | 12.0% | 63 | 27.9% | 188 |
|  | 2 | 73.5% | 225 | 68.5% | 610 | 82.6% | 432 | 59.9% | 403 |
| B10 | 0 | 17.3% | 53 | 16.9% | 150 | 8.8% | 46 | 23.3% | 157 |
|  | 1 | 20.3% | 62 | 22.6% | 201 | 17.6% | 92 | 25.4% | 171 |
|  | 2 | 62.4% | 191 | 60.6% | 539 | 73.6% | 385 | 51.3% | 345 |
| B11 | 0 | 30.4% | 93 | 58.2% | 518 | 34.4% | 180 | 64.0% | 431 |
|  | 1 | 24.5% | 75 | 24.3% | 216 | 26.6% | 139 | 22.6% | 152 |
|  | 2 | 45.1% | 138 | 17.5% | 156 | 39.0% | 204 | 13.4% | 90 |
| B12 | 0 | 11.4% | 35 | 7.9% | 70 | 4.8% | 25 | 11.9% | 80 |
|  | 1 | 28.1% | 86 | 35.8% | 319 | 22.9% | 120 | 42.3% | 285 |
|  | 2 | 60.5% | 185 | 56.3% | 501 | 72.3% | 378 | 45.8% | 308 |
| D1 | 0 | 20.9% | 64 | 23.5% | 209 | 16.1% | 84 | 28.1% | 189 |
|  | 1 | 24.8% | 76 | 26.5% | 236 | 24.1% | 126 | 27.6% | 186 |
|  | 2 | 54.2% | 166 | 50.0% | 445 | 59.8% | 313 | 44.3% | 298 |
| D2 | 0 | 22.5% | 69 | 31.7% | 282 | 23.3% | 122 | 34.0% | 229 |
|  | 1 | 14.4% | 44 | 13.9% | 124 | 10.3% | 54 | 16.9% | 114 |
|  | 2 | 63.1% | 193 | 54.4% | 484 | 66.3% | 347 | 49.0% | 330 |
| D4 | 0 | 22.9% | 70 | 17.1% | 152 | 18.7% | 98 | 18.4% | 124 |
|  | 1 | 28.8% | 88 | 39.3% | 350 | 30.8% | 161 | 41.2% | 277 |
|  | 2 | 48.4% | 148 | 43.6% | 388 | 50.5% | 264 | 40.4% | 272 |
